# Nanoscale x-ray holotomography of human brain tissue with phase retrieval based on multienergy recordings

**DOI:** 10.1117/1.JMI.7.1.013501

**Published:** 2020-01-22

**Authors:** Anna-Lena Robisch, Marina Eckermann, Mareike Töpperwien, Franziska van der Meer, Christine Stadelmann, Tim Salditt

**Affiliations:** a Georg-August-Universität Göttingen, Institut für Röntgenphysik, Göttingen, Germany; b Universitätsmedizin Göttingen, Institut für Neuropathologie, Klinik für Neurologie, Göttingen, Germany; c University of Göttingen, Cluster of Excellence “Multiscale Bioimaging: from Molecular Machines to Networks of Excitable Cells,” Göttingen, Germany

**Keywords:** x-ray phase-contrast microscopy, contrast transfer function, phase retrieval, holography, tomography, virtual histology

## Abstract

X-ray cone-beam holotomography of unstained tissue from the human central nervous system reveals details down to subcellular length scales. This visualization of variations in the electron density of the sample is based on phase-contrast techniques using intensities formed by self-interference of the beam between object and detector. Phase retrieval inverts diffraction and overcomes the phase problem by constraints such as several measurements at different Fresnel numbers for a single projection. Therefore, the object-to-detector distance (defocus) can be varied. However, for cone-beam geometry, changing defocus changes magnification, which can be problematic in view of image processing and resolution. Alternatively, the photon energy can be altered (multi-E). Far from absorption edges, multi-E data yield the wavelength-independent electron density. We present the multi-E holotomography at the Göttingen Instrument for Nano-Imaging with X-Rays (GINIX) setup of the P10 beamline at Deutsches Elektronen-Synchrotron. The instrument is based on a combined optics of elliptical mirrors and an x-ray waveguide positioned in the focal plane for further coherence, spatial filtering, and high numerical aperture. Previous results showed the suitability of this instrument for nanoscale tomography of unstained brain tissue. We demonstrate that upon energy variation, the focal spot is stable enough for imaging. To this end, a double-crystal monochromator and automated alignment routines are required. Three tomograms of human brain tissue were recorded and jointly analyzed using phase retrieval based on the contrast transfer function formalism generalized to multiple photon energies. Variations of the electron density of the sample are successfully reconstructed.

## 1 Introduction

In the hard x-ray regime with multi-keV photon energy E, variations in the index of refraction n=1−δ+iβ are dominated by δ, and the imaginary contribution β accounting for absorption becomes extremely weak. Correspondingly, the main interaction of light exploitable for imaging is related to the local phase shifts of the incoming radiation in proportion to the amount and electron density of the material traversed by the beam. Holographic x-ray phase-contrast imaging^1^ exploits this contrast mechanism due to interference of scattered and transmitted illumination.^2^ Combined with tomography, phase-contrast radiography is a well-established and nondestructive tool to image soft tissue samples in three dimensions.^3^^,^^4^^,^^5^

Depending on the distances between source and detector as well as between source and sample, the interference contrast of the holographic radiographs ranges from enhancing edges comparable to the effect of a high-pass filter to deep holographic fringe structures, which complicate direct interpretation of the projections.^6^ Phase retrieval algorithms for the deep holographic regime exploit sample-dependent constraints and can be implemented based on various iterative schemes, starting from simple alternating projections,^7^ modified hybrid input–output schemes,^8^ or more advanced multiple projections schemes^9^^,^^10^ based on the concept of the relaxed alternating reflections algorithm.^11^ Simultaneous reconstruction of object and probe is also possible, given the sufficient diversity in the data.^12^^–^^14^

In practice, however, linearized single-step phase retrieval is often preferred over computationally costly iterative algorithms, especially three-dimensional (3-D) reconstruction. The most widely used single-step algorithm is based on the contrast transfer function (CTF) as pioneered for x-rays by Cloetens et al.,^15^^–^^17^ Turner et al.,^18^ and Zabler et al.^19^ It relies on the approximations of weak interaction with x-rays so that the complex-valued transmission function can be linearized. Furthermore, the sample is assumed to mainly consist of a single material but with variable density. This is denoted as the homogeneous and weak object assumption. Using this assumption, one can write the Fourier-transformed intensity recorded in the detector plane as a product of an oscillating filter kernel (the CTF) and the Fourier-transformed transmission function of the sample. Phase retrieval is then essentially based on the division of the Fourier-transformed intensities by a filter kernel corresponding to the CTF, followed by an inverse Fourier transform. However, spatial frequencies at the zero crossings of the CTF are not transmitted, and therefore several holographic projections under the same angle but at different defocus positions are recorded for compensation.

Variation of the defocus distance z changes the experimental setup, whereas the x-ray energy is kept constant. Furthermore, geometrical magnification and, at the same time, resolution is decreased by shifting the sample closer to the detector. Typically, holograms are interpolated to the largest magnification and smallest field of view (FOV) before phase retrieval. On top, it is necessary to align the rescaled holograms by subpixel image registration. Unavoidable interpolation and image registration finally will lead to a decrease in resolution^20^.

However, there is a second way of influencing the positions of the minima in the CTF: variation of the wavelength by fixed defocus position. For the direct contrast or edge enhancement regime, this has been explored by Gureyev et al.^21^ For the holographic regime and based on the CTF, a phase retrieval algorithm taking different energies into account was proposed by Kashyap et al.^20^ Furthermore, there exist iterative techniques for the multi-E setting.^22^^,^^23^ Since geometrical magnification remains constant, image registration and interpolation steps are not necessary.^20^^,^^21^ On top, by varying the wavelength, one probes the response of the sample material with respect to different photon energies.

Here, we present a single-step approach based on deconvolving the measured holograms by the CTF to directly access electron “densities” instead of phase shifts. We chose a CTF-based technique in order to keep computation time low and allow for nearly on-the-fly tomographic reconstruction during the experiment.

Measurements were performed at the Göttingen Instrument for Nano-Imaging with X-Rays (GINIX) setup of the P10 beamline at Deutsches Elektronen-Synchrotron (DESY). The instrument is based on a combined optics of elliptical mirrors and an x-ray waveguide positioned in the focal plane for further coherence, spatial filtering, and high numerical aperture.^24^ Upon energy variation, the focal spot is stable enough for imaging, such that automated alignment routines enable quick and uncomplicated variation of the wavelength. Previously, this instrument has been successfully used for nanoscale tomography of unstained brain tissue^3^^,^^5^ bridging the gap between classical histology and 3-D virtual histology. To illustrate the performance of the proposed method, here, we show tomography of tissue taken from a human hippocampus at two different resolutions with a close-up at a blood capillary.

The paper is organized as follows: after a short introduction to the main theoretical concepts in Sec. 2, the method is tested with simulated holograms (Sec. 3). Section 4 reports on the experimental setup used for the measurements described in more detail in Sec. 5. Finally, a summary of the main concepts and results is provided in Sec. 6.

## 2 Theory

The complex index of refraction for x-rays can be written as a function of wavelength and spatial coordinate reflecting the local material properties:

n(ξ,λ)=1−δ(ξ,λ)+iβ(ξ,λ). (1)

The real-valued decrement responsible for the phase shifts associated with x-ray propagation through matter can be written as^25^

$$\delta (\xi ,\lambda )=\frac{\lambda^{2}r_{0}}{2\pi }\rho^{\prime }(\xi ),$$ (2)

with the electron density $\rho^{\prime }(\xi )=Z\rho_{a}(\xi )$, $r_{0}$ denoting the classical electron radius, $\rho_{a}(\xi )$ denoting the atomic number density, and Z denoting the number of electrons, corresponding to the limit of the atomic form factor in forward scattering. If the photoeffect dominates absorption, the imaginary component β(ξ,λ) is given as^25^

$$\beta (\xi ,\lambda )=\frac{\lambda^{2}r_{0}}{2\pi }\rho^{\prime \prime }(\xi ,\lambda )=-\frac{\lambda^{2}r_{0}}{2\pi }\frac{f^{\prime \prime }(\xi ,\lambda )}{Z}\rho^{\prime }(\xi ),$$ (3)

where $\rho^{\prime \prime }(\xi ,\lambda )=-\rho_{a}(\xi )f^{\prime \prime }(\xi ,\lambda )$ includes wavelength-dependent resonance properties of atomic orbitals by the imaginary part of the atomic scattering length $f^{\prime \prime }(\xi ,\lambda )$, which is proportional to the absorption cross section. The absorption component of the refractive index is directly related to the absorption coefficient μ(ξ,λ):

$$\mu (\xi ,\lambda )=\frac{4\pi }{\lambda }\beta (\xi ,\lambda ).$$ (4)

Far from absorption edges and for light materials, μ is proportional to the fourth power of the electron number and inversely proportional to the third power of the photon energy E.^26^ Its wavelength dependency can be approximated as

$$\mu (\xi ,\lambda )=\frac{\mu_{r}}{\lambda_{r}^{3}}\lambda^{3},$$ (5)

with a known index of refraction $\mu_{r}$ at wavelength $\lambda_{r}$ for reference. Expressing $\mu_{r}$ by the corresponding $\beta_{r}$, we find:

$$\mu (\xi ,\lambda )=2r_{0}\rho^{\prime \prime }(\xi ,\lambda_{r})(\frac{\lambda^{3}}{\lambda_{r}^{2}}).$$ (6)

Considering phase shift and absorption, the transmission function of a weak sample can be written as

$$\tau (\xi_{⊥},\lambda )=\exp[-i\lambda D(\xi_{⊥})]\cdot \exp[-\frac{\lambda^{3}}{\lambda_{r}^{2}}B(\xi_{⊥})]\approx 1-i\lambda D(\xi_{⊥})-\frac{\lambda^{3}}{\lambda_{r}^{2}}B(\xi_{⊥}),$$ (7)

where

$$D(\xi_{⊥})≔r_{0}\int \rho^{\prime }(\xi )dz,$$ (8)

$$B(\xi_{⊥})≔r_{0}\int \rho^{\prime \prime }(\xi ,\lambda_{r})dz=\frac{r_{0}}{Z}\int \rho^{\prime }(\xi )f^{\prime \prime }(\xi ,\lambda_{r})dz,$$ (9)

with the spatial coordinate z in the direction of the optical axis. In addition to the photon energy scaling postulated above, if we can assume that the material is characterized by a strict proportionality of the two densities $\rho^{\prime }(\xi )$ and $\rho^{\prime \prime }(\xi ,\lambda_{r})$, we can introduce the constant:

$$C≔\frac{\rho^{\prime \prime }(\xi ,\lambda_{r})}{\rho^{\prime }(\xi )}=\frac{-f^{\prime \prime }(\xi ,\lambda_{r})}{Z}=\frac{\beta_{r}}{\delta_{r}},$$ (10)

where $\beta_{r}$ and $\delta_{r}$ are the absorption and phase-shifting components of the refractive index at a reference wavelength $\lambda_{r}$. This proportionality is in particular warranted for the case of a single homogeneous material, which only varies spatially in its density (and not composition). In this case, it follows that

$$B(\xi_{⊥})=r_{0}C\int \rho^{\prime }(\xi )dz=CD(\xi ),$$ (11)

and correspondingly the transmission function

$$\tau (\xi_{⊥},\lambda )=1-i\lambda D(\xi_{⊥})-\frac{\lambda^{3}}{\lambda_{r}^{2}}CD(\xi_{⊥}).$$ (12)

The Fourier transform (reciprocal coordinates $q_{⊥}$) of $\tau (\xi_{⊥})$ is given as

$$\overset{˜}{\tau }(q_{⊥})=\delta (q_{⊥})-i\lambda \overset{˜}{D}(q_{⊥})-\frac{\lambda^{3}}{\lambda_{r}^{2}}C\overset{˜}{D}(q_{⊥}),$$ (13)

where $\overset{˜}{D}(q_{⊥})$ is the Fourier-transformed $D(\xi_{⊥})$. The Fresnel propagator in paraxial approximation, which accounts for the evolution of an electromagnetic field in free space, is given as^6^

$$\kappa [\chi (q_{⊥})]≔\cos[\chi (q_{⊥})]-i\;\sin[\chi (q_{⊥})],\;\text{where}\;\chi (q_{⊥})≔\frac{\pi }{F}{\left|q_{⊥}\right|}^{2},$$ (14)

with the Fresnel number $F=a^{2}/(\Delta \lambda )$ (smallest resolution element a and longitudinal position Δ along the optical axis) and (unitless) reciprocal coordinates q. Note that constant phase shifts are not considered. The Fresnel number characterizes the imaging regime and can be associated with a single pixel as well as with the full width of the FOV. In the holographic regime ($F_{\mathrm{FOV}}≃1$ and $F_{\text{singlepix}.}\ll 1$) interference fringes resulting from structures of high and low spatial frequencies become visible.

The Fourier-transformed wavefield ${\overset{˜}{\psi }}_{F}(\xi_{⊥})$ at Fresnel number F (assuming uniform plane wave illumination and neglecting constant phase factors) is given as

$${\overset{˜}{\psi }}_{F}(q_{⊥})=\overset{˜}{\tau }\cdot \kappa [\chi (q_{⊥})]=\delta (q_{⊥})-\overset{˜}{D}\{\lambda \;\sin[\chi (q_{⊥})]+\frac{\lambda^{3}}{\lambda_{r}^{2}}C\;\cos[\chi (q_{⊥})]\}\;+i\overset{˜}{D}\{\frac{\lambda^{3}}{\lambda_{r}^{2}}C\;\sin[\chi (q_{⊥})]-\lambda \;\cos[\chi (q_{⊥})]\}.$$ (15)

The Fourier-transformed intensity at Fresnel number F is given as

$${\overset{˜}{I}}_{F}(q_{⊥})={\overset{˜}{\psi }}_{F}(\xi_{⊥})⋆{\overset{˜}{\psi }}_{F}(\xi_{⊥}),$$ (16)

where ⋆ denotes the cross correlation and equivalently

$$I_{F}(\xi_{⊥})=\psi_{F}(\xi_{⊥})\cdot \psi_{F}^{*}(\xi_{⊥})\approx 1-2D(\xi_{⊥})\frac{\lambda^{3}}{\lambda_{r}^{2}}CF^{-1}\{\cos[\chi (q_{⊥})]\}-2D(\xi_{⊥})\lambda F^{-1}\{\sin[\chi (q_{⊥})]\}.$$ (17)

In the last approximation, higher orders of $\lambda \cdot D(\xi_{⊥})$ were neglected (weak object approximation). It follows that

$$D(\xi_{⊥})=F^{-1}(-\frac{F[I_{F}(\xi_{⊥})-1]}{2\{\lambda^{3}C^{\prime }\;\cos[\chi (q_{⊥})]+\lambda \;\sin[\chi (q_{⊥})]\}}),\;\text{where}\;C^{\prime }≔\frac{C}{\lambda_{r}^{2}}=\frac{\beta_{r}}{\delta_{r}\lambda_{r}^{2}}.$$ (18)

Finally, similar to the multiple propagation distances,^19^ we can include multiple wavelengths and a frequency-dependent regularization component $\alpha (q_{⊥})$ in the reconstruction formalism, such that the projected electron density can be approximated as

$$D(\xi_{⊥})=F^{-1}\{-\frac{\underset{i}{\sum }F[I_{i}(\xi_{⊥})-1][\lambda_{i}\;\sin(\chi_{i})+\lambda_{i}^{3}C^{\prime }\;\cos(\chi_{i})]}{\underset{i}{\sum }2{\left[\lambda_{i}\;\sin(\chi_{i})+\lambda_{i}^{3}C^{\prime }\;\cos(\chi_{i})\right]}^{2}+\alpha (q_{⊥})}\}.$$ (19)

The index i refers to the Fresnel number and hence wavelength of measurement i. Following Bartels et al.,^27^ the regularization term $\alpha (q_{⊥})$ is a radially symmetric function decaying toward the center and realized by a step function. The full width at half maximum of the regularization is chosen such that it coincides with the distance between the first two symmetric maxima of the CTF. Its maximum amplitude is set to a small number (typically around $10^{-4}$). By addition to the denominator in the reconstruction formalism, it prevents division by zero at high spatial frequencies. Note that division by zero at low spatial frequencies is already prevented by the cosine term in the CTF.

## 3 Simulation

**Fig. 1:**
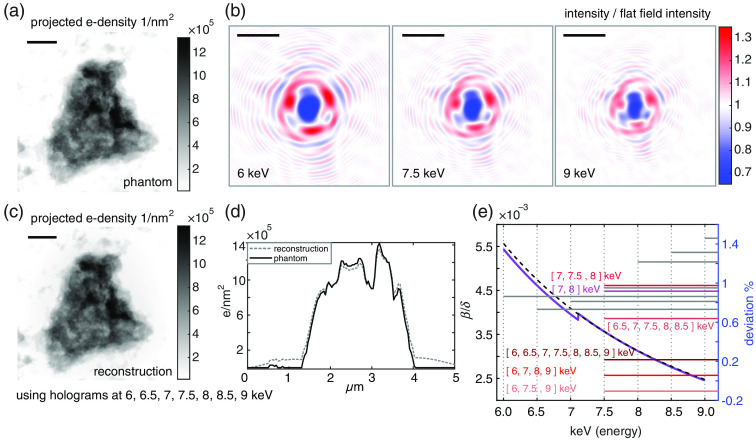
(a) Projected electron density of simulated blood particle; 1  μm scale bar, 38-nm pixel pitch. (b) Holograms from (a) for energies of 6, 7.5, and 9 keV; Fresnel numbers of $2.43\times 10^{-4}$, $3.04\times 10^{-4}$, and $3.65\times 10^{-4}$. Scale bars 5  μm. Holograms were divided by the intensity distribution of the illuminating beam (flat field). (c) Reconstructed projected electron density using seven holograms simulated for [6, 6.5, 7, 7.5, 8, 8.5, 9] keV and Fresnel numbers of $2.43\times 10^{-4}$, $2.64\times 10^{-4}$, $2.84\times 10^{-4}$, $3.04\times 10^{-4}$, $3.24\times 10^{-4}$, $3.45\times 10^{-4}$, $3.65\times 10^{-4}$; 1  μm scale bar. (d) Profile through phantom (a) and reconstruction (c). (e) β/δ-ratio of index of refraction (violet curve) along with continuous model (dashed black curve); relevant energies are highlighted by vertical lines; the mean energy at 7.5 keV was used to estimate the parameter for multienergy reconstructions; horizontal gray lines ending at the respective energies used for reconstruction (single energy reconstructions) show deviations from the expected electron density. Multienergy reconstructions centered around 7.5 keV are marked by colored lines along with the corresponding list of energies.

To check the performance and limitations of the proposed algorithm, a simulation was carried out using a single particle phantom [see Fig. 1(a)] with a volume of 19.8 fL, 3.8  μm maximum, and 1.2  μm mean projected thickness along the optical axis. The particle was assumed to be embedded in air and of uniform material. Its shape was designed based on the projected shape of a 3-D homogeneous cell-like conglomerate.

To match the experimental application, the phantom was designed to mimic the chemical properties of a human erythrocyte. Erythrocytes or red blood cells (RBCs) are responsible for gas exchange and oxygen transport. For this purpose, the protein hemoglobin is used. RBCs consist of 30% to 35% by “weight” of hemoglobin. The remaining components are water (60% to 65%), lipoprotein, and enzymes.^28^ For the following simulation, the phantom was composed of 67.5% water and 32.5% hemoglobin. The molecular formula of hemoglobin is $C_{2932}H_{4724}N_{828}O_{840}S_{8}{\mathrm{Fe}}_{4}$.^29^ The average density of a hemoglobin molecule is $1.335\;{g/\mathrm{cm}}^{3}$.^30^ We model the phase-shifting δ and the absorption component β of the index of refraction as

$$\delta_{\text{blood}}=0.675\cdot \delta_{\text{water}}+0.325\cdot \delta_{\text{hemoglobin}},$$ (20)

$$\beta_{\text{blood}}=0.675\cdot \beta_{\text{water}}+0.325\cdot \beta_{\text{hemoglobin}}.$$ (21)

Values for δ and β are taken from CXRO^31^ and are used to compute an electron density of $375.68{\;\text{electrons}/\mathrm{nm}}^{3}$ [$\rho^{\prime }=2\pi \cdot \delta /(\lambda^{2}r_{0})$], which results in a mean projected electron density of $4.62\cdot 10^{5}\;{\text{electrons}/\mathrm{nm}}^{2}$.

Holograms $I_{\Delta ,k}(\xi_{⊥})$ for the sample–detector distance Δ are computed using the exitwave

$$\tau (\xi_{⊥})=\exp[-ik\int \delta (\xi )-i\beta (\xi )dz],$$ (22)

and the Fresnel propagation operator in paraxial approximation is given as^6^

$$D_{\Delta ,k}[\cdot ]≔\exp(ik\Delta )F^{-1}\exp(-i\Delta \frac{k_{⊥}^{2}}{2k})[\cdot ],$$ (23)

where k=2π/λ is the magnitude of the wave vector $\left(k_{x},k_{y},k_{z}\right)$, such that

$$I_{\Delta ,k}(\xi_{⊥})≔{\left|D_{\Delta ,k}[\tau (\xi_{⊥})]\right|}^{2}.$$ (24)

The phantom (140×140  pixels) was embedded in a large background (3742×3742  pixels), in order to properly sample the Fresnel propagation operator. The free space propagation distance was set to 0.029 m and the pixel pitch to 38 nm. For the selected photon energies, this results in Fresnel numbers of $2.43\cdot 10^{-4}$, $2.64\cdot 10^{-4}$, $2.84\cdot 10^{-4}$, $3.04\cdot 10^{-4}$, $3.24\cdot 10^{-4}$, $3.45\cdot 10^{-4}$, and $3.65\cdot 10^{-4}$, where the smallest resolution element was assumed to be 1 pixel. Figure 1(b) depicts three simulated holograms for energies of 6, 7.5, and 9 keV.

The reconstruction parameter $C^{\prime }$ is given as

$$C^{\prime }(\overset{¯}{\lambda })=\frac{\beta (\overset{¯}{\lambda })}{\delta (\overset{¯}{\lambda })}\frac{1}{{\overset{¯}{\lambda }}^{2}}=\frac{0.675\cdot \beta_{\text{water}}(\overset{¯}{\lambda })+0.325\cdot \beta_{\text{hemoglobin}}(\overset{¯}{\lambda })}{0.675\cdot \delta_{\text{water}}(\overset{¯}{\lambda })+0.325\cdot \delta_{\text{hemoglobin}}(\overset{¯}{\lambda })}\cdot \frac{1}{{\overset{¯}{\lambda }}^{2}},$$ (25)

where $\bar{\lambda }$ is the mean wavelength in case of multiple holograms, or a distinct wavelength in case of a single hologram. For [6, 6.5, 7, 7.5, 8, 8.5, 9] keV, this results in

$$C^{\prime }=[0.1295,0.1287,0.1282,0.1326,0.1320,0.1317,0.1314]\;{\mathrm{nm}}^{-2}$$

for reconstructions from a single hologram and in $C^{\prime }=0.1326\;{\mathrm{nm}}^{-2}$ for reconstructions from several holograms with mean energy of 7.5 keV.

In total, seven holograms were simulated for energies of [6, 6.5, 7, 7.5, 8, 8.5, 9] keV.

Three examples of holograms are shown in Fig. 1(b). Different combinations of holograms all centered around 7.5 keV were used for reconstruction of the electron density. Furthermore, for each energy, reconstructions from single holograms were performed. Electron densities were extracted and deviations from $375.68\;{\text{electrons}/\mathrm{nm}}^{3}$ are computed.

Figure 1(c) shows the reconstructed projected electron density using all seven simulated holograms. Figure 1(d) provides line profiles through phantom (black line) and reconstruction (gray, dashed line). Both agree well despite artifacts at the transition to air. These blurring artifacts are quite common for reconstructions based on the CTF formalism applied to relatively strong samples. However, they vanish for weaker objects. Figure 1(e) finally depicts the ratio between absorption and phase shift (β/δ) (violet curve) as well as the continuous model for this ratio excluding absorption edges and computed on the basis of the mean energy at 7.5 keV. Furthermore, assuming a perfectly known size and shape of the phantom, the reconstructed electron density can be found by division of the projected electron density by the projected thickness: to extract the mean reconstructed electron density, projected electron densities were divided by the projected thickness of the phantom and an average electron density value was computed using the centered 40×40  pixels. For reconstructions from a single hologram, deviations about 1% from the expected electron density were found. Regarding combinations of holograms, it turned out that reconstructions closely match expectations (deviations are about 0.2%) for a few holograms spanning a wide energy range, in comparison to the same number of holograms spanning a small energy range. Even small discontinuities (weak absorption edge) and strong phase shifts still allow retrieving reasonable values of the electron density.

## 4 Experimental Setup

**Fig. 2:**
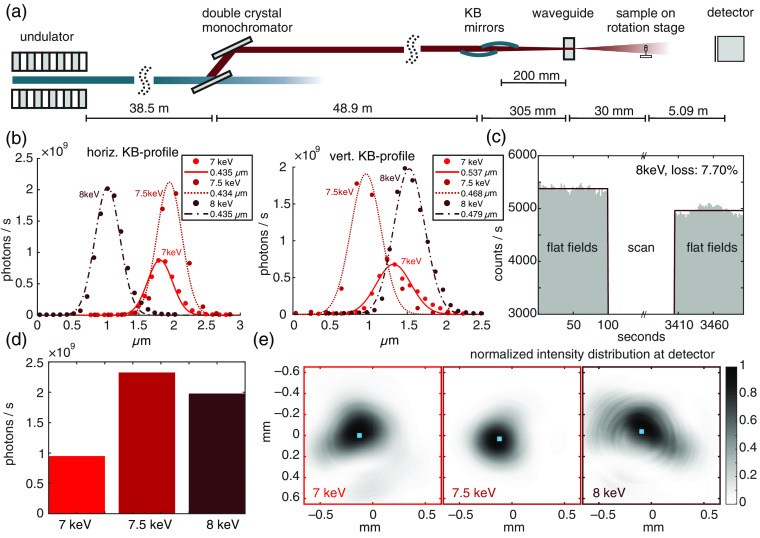
(a) Layout of the P10 beamline at DESY for nanoscale tomography. (b) Horizontal and vertical profiles of the KB focus at energies of 7, 7.5, and 8 keV. (c) Stability of illumination during a tomography scan at 8 keV. (d) Transmission of the waveguide at 7, 7.5, and 8 keV. (e) Two-dimensional normalized intensity profiles of the beam for 7, 7.5, and 8 keV measured at 5.12 m behind the waveguide exit and with highlighted position of the center of mass for each profile.

Experiments were performed at the GINIX setup of beamline P10 at DESY.^24^ The GINIX setup supports three main applications: scanning nanobeam diffraction, coherent diffractive imaging, and holographic imaging. In this experiment, the last modality was used. A schematic sketch showing the main components of the beamline layout, including the endstation, is shown in Fig. 2(a). At a 38.5-m distance from the undulator, a fixed-offset double-crystal Si(111) monochromator (DCM) is installed to select photon energies in the medium–hard energy range. Next, 48.9 m behind the DCM, a fixed curvature Kirkpatrick–Baez (KB) mirror system is used as focusing device with its focal spot at a 87.7-m distance from the undulator. For holographic imaging, an x-ray waveguide mounted on a hexapodsystem (SmarAct) is installed in the common focal plane of the KB mirrors.

The waveguide fulfills two purposes: first, it further concentrates the radiation and hence enables a highly divergent cone-beam illumination, and second, it works as a coherence filter and enables illuminating with highly coherent spherical wavefronts.^32^^–^^34^ For this experiment, a channel waveguide in silicon, fabricated by e-beam lithography and wafer bonding was used.^34^ The waveguide channel used for the presented experiments was 90 (horz.)×70  nm (vert.) and cut to a length of 1 mm. The flux at the waveguide exit ranged between $10^{8}$ and $10^{9}\;\text{photons}/s$. Since waveguides and KB mirrors are both reflective optics, they are comparably insensitive to variation of the photon energy and a slight realignment is enough to ensure sufficient flux after energy change.^24^ The full width at half maximum for the KB probe as well as the position of the focal spot in the plane perpendicular to the optical axis was determined by scanning the waveguide through the KB focus for different energies and using a single-photon counting detector (Dectris, Pilatus 300k). Upon energy change between 7 and 8 keV, the full widths at half maximum for the KB beam varied between 536.9 and 468.4 nm for the vertical direction, and between 435.2 and 434.5 nm in the horizontal direction, whereas the maximum change in vertical position was 569 nm and in horizontal position 915 nm [see Fig. 2(b)]. The total flux behind the waveguide measured by the single-photon counting detector was $0.95\times 10^{9}\;\text{photons}/s$ at 7 keV, $2.32\times 10^{9}\;\text{photons}/s$ at 7.5 keV, and $1.98\times 10^{9}\;\text{photons}/s$ at 8 keV [see Fig. 2(d)]. Two-dimensional intensity profiles of the waveguide probes at the detector along with the respective positions of the center of mass are depicted in Fig. 2(e). For all three energies, a relatively homogeneous illumination with only slight change in position of the center of mass was observed. After each energy change, an automated alignment routine was used for optimally positioning the waveguide. This was done by alternately scanning the waveguide horizontally and vertically through the focal plane of the KB until maximum throughput of photons was reached.

Downstream the waveguide, the tomography sample stage was installed. For the current experiments, the distance between waveguide exit and sample stage was set to 30 mm. Subsequently, x-rays propagate in free space for 5.09 m where the intensity was recorded by a 2×2  k sCMOS camera with a gadolinium oxysulphide scintillator and 6.5  μm pixel pitch d (Photonic Science).

Owing to the cone-beam illumination and the absence of any optics in the beampath, the recorded images are magnified holographic intensity distributions resulting from divergence and interference of transmitted and scattered light. The geometric magnification M of the projections depends on the distance between waveguide exit and sample ($z_{01}$) and the distance between sample and detector ($z_{12}$) and is given as $M=(z_{01}+z_{12})/z_{01}$. Following the Fresnel scaling theorem,^6^ magnified intensity recordings can be treated as holograms resulting from a parallel-beam illumination in an effective, scaled coordinate system. This means that the free space propagation distance of the exitwave $z_{12}$ is replaced by $z_{\mathrm{eff}}=z_{12}/M$ and the effective pixel pitch is $d_{\mathrm{eff}}=d/M$. For the described setting, geometric magnification is ×170.69, resulting in an effective propagation distance of 29.82 mm and an effective pixel pitch of 38.08 nm. Owing to the magnifying cone-beam setting, the FOV covered by a single projection was $78\times 78\;\mu m^{2}$ (2048×2048 detector pixels).

The particular sample used here for the proof of concept of multi-E holotomography was scanned as part of a biomedical study (approved by the ethics committee of the University Medical Center Göttingen) devoted to 3-D patho-histology of Alzheimer’s disease. A formalin-fixed, paraffin-embedded tissue specimen of 1 mm in diameter (autopsy from human hippocampus) was kept inside a polyimide (Kapton) tube.

For a full tomography scan, the sample was rotated between 0 deg and 180 deg in 1000 steps. At each position, an inline hologram was recorded. This procedure was repeated for 7, 7.5, and 8 keV illumination energies. The exposure times were 3 s (7 keV) and 2 s (7.5 and 8 keV). During scanning, the total flux remained almost constant. Figure 2(c) reports only a slight decrease in photons, which can be attributed to small drifts of the waveguide optics. Since neither projection shows the entire sample, all of the recorded holograms are the so-called truncated projections, and reconstruction faces the additional challenge of region-of-interest tomography.

## 5 Experiment

**Fig. 3:**
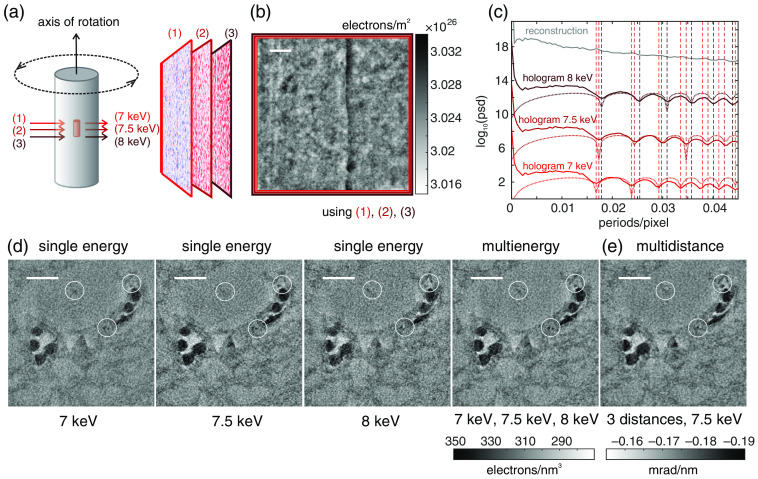
(a) Sketch of the sample (brain tissue in polyimide tube) with highlighted region of interest (red cylinder). For each angular position and each of the three energies, separate holographic projections were recorded. (b) Example of a projection after reconstruction of the projected electron density using three recordings at different energies (1)–(3); scale bar 10  μm. (c) Angular averaged power spectral density (psd) for holograms recorded at 7, 7.5, and 8 keV and Fresnel numbers of $2.84\times 10^{-4}$, $3.01\times 10^{-4}$, $3.18\times 10^{-4}$ along with the phase CTFs and the power spectral density of the reconstruction (shifted for clarity). Dashed vertical lines indicate positions of minima of the transfer functions. (d) Reconstructed slices after tomography using reconstructed projections from holograms recorded at 7 keV only, at 7.5 keV only, and at 8.0 keV only and reconstruction using all holograms. The mean gray values were shifted to the mean expected electron density of paraffin. Differences in image quality can be seen in the highlighted regions. Scale bar 10  μm. (e) Tomographic reconstruction using the classical CTF approach with distance variation. Scale bar 10  μm.

Figure 3(a) shows the configuration of multi-E tomography. The sample (fixed tissue in paraffin and polyimide tube) was rotated around the vertical axis. For each distinct energy and at discrete angles between 0 deg and 180 deg, a holographic projection was recorded. For reconstruction, a crucial step is a proper choice of the parameter $C^{\prime }$. For this special case, we faced two main issues: first, since the measurements are truncated projections and since the diameter of the beam is smaller than the sample, there is no interference with an empty reference beam (x-rays that do not pass through the sample). Reconstructed phase shifts (and so reconstructed electron densities) can, therefore, only be interpreted as relative quantities with respect to an unknown offset. Second, in order to estimate a meaningful value for $C^{\prime }$, assumptions on the material composition of the sample have to be made. We assumed the sample to consist mainly of paraffin, which we modeled by the chemical formula $C_{30}H_{62}$ with density $\rho_{m}$ of $0.9\;{g/\mathrm{cm}}^{3}$ and an electron density $\rho_{e}$ of about

$$\rho_{e}=\frac{\rho_{m}}{MN_{a}N_{e}}=310.25\;\text{electrons}/{\mathrm{nm}}^{3},$$ (26)

where $N_{a}$ is the Avogadro’s constant, M is the molar mass, and $N_{e}$ is the number of electrons.

Holograms have been recorded at 7, 7.5, and 8 keV (Fresnel numbers $2.84\times 10^{-4}$, $3.01\times 10^{-4}$, $3.18\times 10^{-4}$). At a mean energy of 7.5 keV (wavelength of $\overset{¯}{\lambda }=0.164\;\mathrm{nm}$), the phase-shifting component of the index of refraction for paraffin is $\delta =3.810\cdot 10^{-6}$ and the absorption component is $\beta =5.467\cdot 10^{-9}$. Therefore, the parameter $C^{\prime }$ can be estimated as

$$C^{\prime }(\overset{¯}{\lambda })=\frac{\beta (\overset{¯}{\lambda })}{\delta (\overset{¯}{\lambda })}\frac{1}{{\overset{¯}{\lambda }}^{2}}=0.0534\;{\mathrm{nm}}^{-2}.$$ (27)

Similarly, parameters for $C^{\prime }$ can be found for single-energy reconstructions at the respective energies: $0.0537\;{\mathrm{nm}}^{-2}$ (7 keV), $0.0534\;{\mathrm{nm}}^{-2}$ (7.5 keV), and $0.0531\;{\mathrm{nm}}^{-2}$ (8 keV).

Figure 3(b) shows the reconstruction of one angular position using holograms recorded at 7, 7.5, and 8 keV and setting the reconstruction parameter to $C^{\prime }=0.0534\;{\mathrm{nm}}^{-2}$. Even though the range spanned by the used energies is quite narrow, it is sufficient to compensate minima in the power spectral density of the reconstruction: whereas distinct minima can be seen in the angular averaged power spectrum of the single holograms, they do not remain in the final power spectrum of the reconstructed electron density map [see Fig. 3(c)]. Further analysis of the power spectral densities of the three holograms showed that noise dominated at 0.147 periods/pixel (7 keV), 0.166 periods/pixel (7.5 keV), and 0.159 periods/pixel (8 keV).

After retrieval of the projected electron density, the next step is to perform tomographic reconstruction by filtered backprojection. First, in order to reduce tomographic sampling artifacts, the single sinograms were interpolated by a factor of 2 in the angular direction. Second, to remove artifacts resulting from truncated projections, the sinograms were extended by repeating the outermost pixels in positive and negative spatial directions. Third, a wavelet-based filter was used to remove ring artifacts.^35^ Finally, the mean gray value of the slices reconstructed by filtered backprojection was shifted to the expected electron density of paraffin.

Figure 3(d) shows four tomographic reconstructions, i.e., horizontal slices through four tomographic volumes at comparable positions: One for each of the three energies (7, 7.5, and 8 keV) and for common electron density retrieval using all holograms. The single slices are maximum intensity projections, i.e., compositions of the maximum gray values along a depth of 11 voxels. The last reconstruction (multi-E reconstruction) reveals most details and less noise [see marked regions by white circles in Fig. 3(d)].

In Fig. 3(e), a tomographic slice (minimum intensity projection over 11 voxels) from classical three-distance CTF-based phase retrieval is shown (Fresnel numbers: $3.06\times 10^{-4}$, $3.16\times 10^{-4}$, $3.47\times 10^{-4}$; energy: 7.5 keV; and distances source-sample: 30, 31, and 34 mm). Note that resolution and contrast are rather comparable to the respective single energy reconstruction for 7.5 keV while the multi-E tomogram reveals slightly more details. This difference may be attributed to interpolation and image registration steps prior to multidistance phase retrieval. Hence, in accordance with Kashyap et al.,^20^ we conclude that multi-E CTF-based imaging is at least as good as the multidistance approach.

**Fig. 4:**
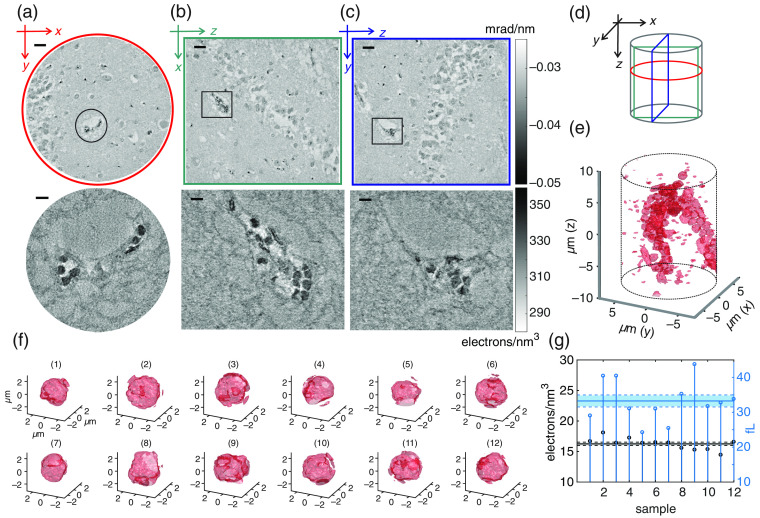
(a)–(c) Slices through the tomographic volume of a large FOV scan (top) and the multi-E high-resolution scan (bottom). Scale bars for the large FOV: 25  μm, scale bars for the small FOV: 5  μm. (d) Illustration of the spatial orientation of the slices shown in (a)–(c). (e) Tomographic high-resolution volume after segmentation of the RBCs. (f) Segmentation of single RBCs used for analysis of volume and electron density with respect to the electron density of paraffin. (g) Electron density difference with respect to paraffin and volume of the selected cells shown in (f).

Next, the tomographic reconstruction will be put in a broader context. Figures 4(a)–4(c, top) show tomographic slices of a three-distance scan with larger FOV (Fresnel numbers: $14.69\times 10^{-4}$, $15.45\times 10^{-4}$, $16.62\times 10^{-4}$; energy 8 keV; and distances source-sample: 137, 142, and 152 mm). Figures 4(a)–4(c), bottom) show the multi-E high-resolution scan covering a small FOV highlighted in the overview scan. Figure 4(d) depicts the volume geometry to illustrate the spatial orientation of the slices shown in Figs. 4(a)–4(c). In the overview scan, the dentate gyrus in the hippocampus is visible along with spread out neurons and blood capillaries. We focus on the highlighted blood capillary for which the close-up multi-E scans were recorded, and choose the highly contrasted RBCs in the vessel as a testbed for quantification of size, shape, and location. To this end, a threshold-based segmentation algorithm with repetitive image erosion and dilation steps was used. Figure 4(e) shows the segmented cells inside the tomographic volume. Twelve cells were chosen in Fig. 4(f) and separately analyzed regarding their volume and electron density with respect to the mean reconstructed electron density. Figure 4(g) displays the volume of each of the particles with a mean value of 33.25±1.72  fL, and a mean reconstructed excess electron density (with respect to paraffin) of $16.26\pm 0.28\;{\text{electrons}/\mathrm{nm}}^{3}$. The significant shrinking of the RBCs with respect to the physiological volume ranging between 80 and 100 fL can be attributed to sample preparation as well as postmortem deformations, which also manifest themselves in the distorted shapes of RBCs. Note that these shrinkage artifacts observed here for the RBCs are much more pronounced than the average histological shrinking of tissue, which is typically between 8% and 20% in each direction.^36^ At the same time, the measured excess electron density (with respect to paraffin) is lower than the value of $39\;{\text{electrons}/\mathrm{nm}}^{3}$, which we would obtain by a “naïve” model, replacing water by paraffin based on the volume fraction and stoichiometry given in Sec. 3 [Eqs. (20) and (21)].

## 6 Conclusion

In this work, an approach of direct electron density reconstruction based on the CTF formalism and holograms recorded at multiple wavelengths was presented, demonstrated by simulations and experimental data representative for a relevant 3-D histopathology study.

We showed that suitable instrumental settings, including a fixed-offset DCM and nondispersive focusing optics as given at beamline P10/GINIX, allow for a convenient experimental realization of multienergy recordings.

Phase-contrast x-ray tomography opens up not only an attractive 3-D extension of current histology but also a contrast mechanism based on electron density differences. To exploit these opportunities for quantitative studies of tissue, CTF-based phase retrieval is well suited based on its fast implementation. The inherent linearization (weak object constraint) and the coupling of phase and absorption (single material or homogeneous object constraint) seem to be well warranted for unstained soft tissue samples at multi-keV radiation.^5^ Rather than the conventional multidistance CTF recordings, we have circumvented underdetermined data associated with the zero crossings of the CTF by varying the photon energy. Extending earlier work, in particular the multi-E CTF demonstration by Kashyap et al.,^20^ we have shown that high image quality can be reached even at high magnification in a cone-beam setting. This is of particular interest since registration of multidistance CTF datasets can be problematic when changing magnification as in a defocus series. Furthermore, multi-E CTF combines data diversity by changing the Fresnel number F with controlled changes in the interaction parameters of the sample, which changes the relative weight of absorption and phase simultaneously for a range of spatial frequencies.

In this work we have assumed simple scaling relations of $\delta \propto \lambda^{2}$ and of $\beta \propto \lambda^{4}$, but future extensions of the data acquisition (multienergy and multidistance) as well the reconstruction scheme can be devised to account for anomalous effects near absorption edges, opening up a means for elemental contrast. For pure absorption-based CT, energy selective reconstructions allow material characterization by splitting the attenuation coefficient in a contribution due to the photoelectric effect and a contribution due to Compton scattering.^37^ Similarly, for inline holographic phase-contrast imaging at comparably low photon energies, in an extended scheme with independent β(λ) and δ(λ) material specific segmentation of the 3-D reconstruction should be possible. To this end, implementation of iterative procedures and decoupling the absorption and phase-shifting image properties as well as the design of suitable well-characterized tomographic phantoms will be important next steps. For human neuronal tissue, local Fe accumulations may reach the minimum concentrations required to reconstruct iron maps or “iron-weighted images.” Specific heavy-atom labels with carefully chosen absorption edges may also be interesting targets for future studies.
